# Investigation of dose-rate effects and cell-cycle distribution under protracted exposure to ionizing radiation for various dose-rates

**DOI:** 10.1038/s41598-018-26556-5

**Published:** 2018-05-29

**Authors:** Yusuke Matsuya, Stephen J. McMahon, Kaori Tsutsumi, Kohei Sasaki, Go Okuyama, Yuji Yoshii, Ryosuke Mori, Joma Oikawa, Kevin M. Prise, Hiroyuki Date

**Affiliations:** 1 Graduate School of Health Sciences, Hokkaido University, Sapporo, 060-0812 Japan; 2 Centre for Cancer Research & Cell Biology, Queen’s University Belfast, Belfast, BT9 7AE UK; 3 Faculty of Health Sciences, Hokkaido University, Sapporo, 060-0812 Japan; 4 Faculty of Health Sciences, Hokkaido University of Science, Sapporo, 006-8585 Japan; 5 Biological Research, Education and Instrumentation Center, Sapporo Medical University, Sapporo, 060-8556 Japan

## Abstract

During exposure to ionizing radiation, sub-lethal damage repair (SLDR) competes with DNA damage induction in cultured cells. By virtue of SLDR, cell survival increases with decrease of dose-rate, so-called dose-rate effects (DREs). Here, we focused on a wide dose-rate range and investigated the change of cell-cycle distribution during X-ray protracted exposure and dose-response curves via hybrid analysis with a combination of *in vitro* experiments and mathematical modelling. In the course of flow-cytometric cell-cycle analysis and clonogenic assays, we found the following responses in CHO-K1 cells: (1) The fraction of cells in S phase gradually increases during 6 h exposure at 3.0 Gy/h, which leads to radio-resistance. (2) Slight cell accumulation in S and G_2_/M phases is observed after exposure at 6.0 Gy/h for more than 10 hours. This suggests that an increase of SLDR rate for cells in S phase during irradiation may be a reproducible factor to describe changes in the dose-response curve at dose-rates of 3.0 and 6.0 Gy/h. By re-evaluating cell survival for various dose-rates of 0.186–60.0 Gy/h considering experimental-based DNA content and SLDR, it is suggested that the change of S phase fraction during irradiation modulates the dose-response curve and is possibly responsible for some inverse DREs.

## Introduction

The impact of ionizing radiation on mammalian cells depends significantly on the particle fluence of radiation per unit of time, so called dose-rate effects (DREs)^1^. During protracted irradiation at lower dose-rates, induction of toxic DNA lesions along the particle track competes with DNA damage repair, which leads to reduced cell-killing^2^. However, in recent decades, increased induction of mutation or chromosomal aberrations^3,4^ and enhancement of cell-killing in a lower dose-rate range of 10–100 cGy/h^5^ have been reported, so-called “inverse dose-rate effects (IDREs)”. Under low-dose exposure, mammalian cells exhibit hyper radio-sensitivity (HRS) to doses with <30 cGy which is believed to result from failure to arrest in G_2_^6,7^, whilst intercellular signalling has also been reported to have the potential capacity to enhance cell-killing^8,9^. Although the involvement of the cellular signalling in IDREs has been presumed, the underlying mechanism of IDREs remains unclear. Re-evaluation of the DREs on cell survival including IDREs is a crucial issue from the standpoints of radiation therapy and radiation protection^10^.

The sparing effects of cell-killing under a lower dose-rate can be explained by sub-lethal damage repair (SLDR) during irradiation^2^. SLDR during exposure also contributes to a decrease of the quadratic component in high-dose ranges^2^. Under the confluent condition of cells represented as plateau phase (similar to conditions in tissue)^11^, the cell-cycle distribution is mainly composed of cells in G_1_ phase. There have been some reports that the fraction of cells in G_2_/M phase gradually increases during protracted irradiation, i.e., at 60 cGy/h in tumour cell line of T98G (derived from human glioblastoma multiforma) and U373MG (derived from human glioblastoma astrocytoma) and at 100 cGy/h in CHO-K1 (derived from Chinese Hamster ovary)^5,12^. As reported in our previous study, the fractionated regimen of 1 Gy per fraction at every 1 h time interval, which is similar to continuous exposure at 1.0 Gy/h, was used to discuss the cell-cycle change^12^. In such an irradiation, the accumulation in G_2_/M phase under lower dose-rate may be associated with higher radio-sensitivity^12^. In this regard, radio-sensitivity during exposure can be potentially modulated by not only intercellular signalling as suspected recently but also changes in cell-cycle distribution^13,14^ including cell multiplication^15,16^. Thus, it is necessary to investigate the change for various dose-rates at the level of *in vitro* experiments.

From the viewpoint of estimating dose-response curves, the curves can be described in general by taking account of SLDR rate deduced from a split-dose cell recovery^17,18^. According to the previous reports^2,17,18^, the repair half-time of SLD is cell type and cell condition specific, e.g., 0.985 h in CHO cells in plateau phase. The linear-quadratic (LQ) model with Lea-Catcheside time factor^19^ or microdosimetric-kinetic (MK) model^17^ have been used to analyse cell survival considering SLDR during irradiation at the level of cell populations. However, recent model analysis using the MK model suggests that rate of SLDR depends on dose-rate, in which the SLDR rate decreases as dose-rate lowers^20^. This interpretation may be linked to cell-cycle changes, but there is currently no report with evidence to support that SLDR changes depending on dose-rate. Thus, the interest in this study is directed to the consideration of SLDR depending on dose-rate associated with experimentally determined cell-cycle distribution during irradiation.

In this study, we used the Chinese Hamster Ovary (CHO)-K1 cell line that does not exhibit low-dose HRS^21^ and newly observed the dose-rate dependence of cell survival in relation to the change of cell-cycle distribution during irradiation at 3.0 Gy/h (1.5 Gy per fraction at every 30 min interval, 24 fractions) and 6.0 Gy/h (2.0 Gy per fraction at every 20 min interval, 36 fractions) in addition to our previous data at 1.0 Gy/h (1.0 Gy/fr at every 1 h time interval, 12 fractions). Combined with previous cell responses for 0.186, 1.0, 1.5, 10.8, 18.6, 60.0 Gy/h, here we re-evaluated the radio-sensitivity at the endpoint of cell survival and mean inactivation dose. Finally we show that the changes of radio-sensitivity in dose-response curves under continuous irradiation can be explained by changes of SLDR rate due to an increase of cells in S phase.

## Model Overview

### Methodology of Cell-Killing Model

In order to determine a fractionated regimen equivalent to a long-term continuous exposure and to predict DREs on cell survival, we used the microdosimetric-kinetic (MK) model for continuous irradiation^17^. The MK model has been developed based on microdosimetry^22^ and popular theory of damage behaviour^23,24^ by comparing with several experimental data so far^2,12,17,18,20,25–29^. In this study, we further developed the MK model so as to consider change of DNA amount per nucleus and SLDR rate during irradiation, hereafter called the “integrated microdosimetric-kinetic (IMK) model”.

Briefly, the MK model^17^ subdivides the cell nucleus into a lot of micro-order territories (so-called domains) so as to incorporate microdosimetry^22^. The shape of the domains is for simplicity defined as a sphere with radius from 0.5 to 1.0 μm^17,20^ and the local energy deposition along radiation particle track can be evaluated in terms of tissue equivalent proportional counter (TEPC) measurements^27^ or Monte Carlo simulations^27,30^. DNA damage which may be toxic to the cell is represented as a potentially lethal lesion (PLL), induced in a domain packaging DNA amount of *g* (kg) along the particle track with local dose deposition (as in Gy) per domain *z*. The DNA lesion (PLL) can gradually transform into lethal lesion (LL) or be repaired until no PLL remain:

(i) A PLL may transform into a lethal lesion (LL) via a first-order process at a constant rate *a* (h^−1^)
(ii) Two PLLs may interact and transform into a lethal lesion (LL) via a second-order process at a constant rate *b*_d_ (h^−1^)
(iii) A PLL may be repaired via a first-order process at constant rate *c* (h^−1^).

The rate equation for the number of PLLs per domain after acute exposure *x*_d_(*t*) is given by

1 $$\begin{array}{rcl}\frac{{\rm{d}}}{{\rm{d}}t}{x}_{{\rm{d}}}(t) & = & -(a+c){x}_{{\rm{d}}}(t)-{\rm{2}}{b}_{{\rm{d}}}{x}_{{\rm{d}}}{(t)}^{2},\\  & \cong \, & -(a+c){x}_{{\rm{d}}}(t){\rm{.}}\,\because (a+c){x}_{{\rm{d}}}(t)\gg 2{b}_{{\rm{d}}}{x}_{{\rm{d}}}{(t)}^{2}\end{array}$$

This can be solved as exponential function expressed by

2 $${x}_{{\rm{d}}}(t)={k}_{{\rm{d}}}gz{e}^{-(a+c)t},\,\because {x}_{{\rm{d}}}({\rm{0}})={k}_{{\rm{d}}}gz,$$

where *k*_d_ is the PLL induction coefficient per DNA amount and imparted energy in the domain.

Let us consider a continuous exposure to a cell population with dose-delivery time *T* (h) and dose-rate $\dot{D}$ (Gy/h). To model this, during the dose-delivery, specific energy (*z*_1_, *z*_2_, …, *z*_N_) is discontinuously deposited in a domain with amount of DNA (*g*_1_, *g*_2_, …, *g*_N_) at each sub-section of dose-delivery time ([0, *ΔT*), [*ΔT*, 2*ΔT*), …, [(*N* − 1)*ΔT*, *NΔT*)), thus we obtain the relation of *T* = *N*Δ*T*, where *N* is the number of sub sections in total dose-delivery time *T*^12,17^. In this study, we newly assumed that the rate of SLDR (*c*_1_, *c*_2_, …, *c*_N_) changes at each sub-section of *T* ([0, *ΔT*), [*ΔT*, 2*ΔT*), …, [(*N* − 1)*ΔT*, *NΔT*)). Thus, the number of PLLs per domain *x*_*d*_(*t*) can be expressed based on Eq. (2) as

3 $$\begin{array}{rcl}{x}_{{\rm{d}}}(t) & = & {k}_{{\rm{d}}}{g}_{1}{z}_{1}{e}^{-(a+{c}_{1})t}\\  &  & \times \{t|0\le t < \Delta T\}\\ {x}_{{\rm{d}}}(t) & = & {\sum }_{n=1}^{2}{k}_{{\rm{d}}}{g}_{n}{z}_{n}{e}^{-(a+{c}_{n})[t-(n-1){\Delta }T]}\\  &  & \times \{t|\Delta T\le t < 2\Delta T\}\\ \vdots  &  & \\ {x}_{{\rm{d}}}(t) & = & {\sum }_{n=1}^{N-1}{k}_{{\rm{d}}}{g}_{n}{z}_{n}{e}^{-(a+{c}_{n})[t-(n-1){\Delta }T]}\\  &  & \times \{t|(N-2)\Delta T\le t < (N-1)\Delta T\}\\ {x}_{{\rm{d}}}(t) & = & {\sum }_{n=1}^{N}{k}_{{\rm{d}}}{g}_{n}{z}_{n}{e}^{-(a+{c}_{n})[t-(n-1){\Delta }T]}.\\  &  & \times \{t|(N-1)\Delta T\le t\}\end{array}$$

Whilst PLLs’ induction competes with SLDR during dose-delivery, LLs gradually increase according to the next rate equation as

4 $$\frac{{\rm{d}}}{{\rm{d}}t}{w}_{{\rm{d}}}=a{x}_{{\rm{d}}}(t)+{b}_{{\rm{d}}}{x}_{{\rm{d}}}{(t)}^{2},$$

where *w*_d_ is the number of LLs per domain. Solving Eq. (4) for the time dependent repair in Eq. (3), we can obtain the accumulated number of LLs per domain as,

5 $$\begin{array}{ccc}{w}_{{\rm{d}}} & =\, & \,{\sum }_{n=1}^{N}({A}_{n}{g}_{n}{z}_{n})+{\sum }_{n=1}^{N}({B}_{n}{{g}_{n}}^{2}{{z}_{n}}^{2})\,\\  &  & +2{\sum }_{n=1}^{N-1}{\sum }_{m=n+1}^{N}[{B}_{nm}{g}_{n}{g}_{m}{z}_{n}{z}_{m}{e}^{-(m-n)(a+{c}_{n}){\rm{\Delta }}{T}}],\end{array}$$

where

6a $${A}_{n}=\frac{a{k}_{{\rm{d}}}}{(a+{c}_{n})}\cong \frac{a{k}_{{\rm{d}}}}{{c}_{n}}$$

6b $${B}_{n}=\frac{{b}_{{\rm{d}}}{{k}_{{\rm{d}}}}^{2}}{2(a+{c}_{n})}\cong \frac{{b}_{{\rm{d}}}{{k}_{{\rm{d}}}}^{2}}{2{c}_{n}}$$

6c $${B}_{nm}=\frac{{b}_{{\rm{d}}}{{k}_{{\rm{d}}}}^{2}}{(a+{c}_{n})+(a+{c}_{m})}=\frac{2{B}_{n}(a+{c}_{n})}{(a+{c}_{n})+(a+{c}_{m})}\cong \frac{2{B}_{n}{c}_{n}}{{c}_{n}+\,{c}_{m}}$$

In Eq. (6), the value of *a* is a few percent of *c*_*n*_^17,20^, thus (*a* + *c*_*n*_) can be simply approximated by *c*_*n*_. Let <*w*_d_> and <*w*>_T_ be the average number of lethal lesions per domain and the average number of LLs per nucleus, respectively. Considering the mean dose per nucleus <*z*_*n*_> and the mean amount of DNA per nucleus <*G*_*n*_> at the timing of *t* = (*n* *−* 1)*ΔT* and assuming that the absorbed dose rate is constant (<*z*_1_> = <*z*_2_> = , …, = <*z*_*n*_> = $\dot{D}$*ΔT*), <*w*>_T_ is expressed as

7 $$\begin{array}{rcl}\,{w}_{{\rm{T}}} & = & \langle p{w}_{{\rm{d}}}\rangle \\  & = & {\sum }_{n=1}^{N}({A}_{n}p{\int }_{0}^{\infty }{g}_{n}{f}_{g}({g}_{n}){\rm{d}}{{g}}_{n}{\int }_{0}^{\infty }{z}_{n}{f}_{z}({z}_{n}){\rm{d}}{{z}}_{n})\\  &  & +\,\,{\sum }_{n=1}^{N}({B}_{n}p{\int }_{0}^{\infty }{{g}_{n}}^{2}{f}_{g}({g}_{n}){\rm{d}}{{g}}_{n}{\int }_{0}^{\infty }{{z}_{n}}^{2}{f}_{z}({z}_{n}){\rm{d}}{{z}}_{n})\\  &  & +\,\,{\rm{2}}{\sum }_{n=1}^{N-1}{\sum }_{m=n+1}^{N}[{B}_{{nm}}p{\int }_{0}^{\infty }{g}_{n}{f}_{g}({g}_{n}){\rm{d}}{{g}}_{n}{\int }_{0}^{\infty }{z}_{n}{f}_{z}({z}_{n}){\rm{d}}{{z}}_{n}\\  &  & \,\times {\int }_{0}^{\infty }{g}_{m}{f}_{g}({g}_{m}){\rm{d}}{{g}}_{m}{\int }_{0}^{\infty }{z}_{m}{f}_{z}({z}_{m}){\rm{d}}{{z}}_{m}{e}^{-(m-n)(a+{c}_{n}){\Delta }T}],\\ {\langle w\rangle }_{{\rm{T}}} & = & {\sum }_{n=1}^{N}[({A}_{n}\langle {G}_{n}\rangle +\gamma \frac{{B}_{n}}{p}\langle {{G}_{n}}^{2}\rangle )\dot{D}{\Delta }T+\frac{{B}_{n}}{p}\langle {{G}_{n}}^{2}\rangle {(\dot{D}{\Delta }T)}^{2}]\\  &  & +2{\sum }_{n=1}^{N-1}{\sum }_{m=n+1}^{N}[\frac{{B}_{{nm}}}{p}\langle {G}_{n}\rangle \langle {G}_{m}\rangle {e}^{-(m-n)(a+{c}_{n}){\Delta }T}]{(\dot{D}{\Delta }T)}^{2},\end{array}$$

where

8a $$\dot{D}{\Delta }T=\langle {z}_{n}\rangle ={\int }_{0}^{\infty }{z}_{n}{f}_{z}({z}_{n}){\rm{d}}{{z}}_{n},$$

8b $${(\dot{D}{\Delta }T)}^{2}+\gamma \dot{D}{\Delta }T={\langle {z}_{n}\rangle }^{2}+\frac{{y}_{D}}{{\rm{\rho }}{\rm{\pi }}{r}_{{\rm{d}}}^{\,2}}\langle {z}_{n}\rangle ={\int }_{0}^{\infty }{{z}_{n}}^{2}{f}_{z}({z}_{n}){\rm{d}}{z}_{n},$$

8c $$\langle {G}_{n}\rangle =p{\int }_{0}^{\infty }{g}_{n}\,{f}_{g}({g}_{n}){\rm{d}}{g}_{n},$$

8d $$\langle {{G}_{n}}^{2}\rangle ={p}^{2}{\int }_{0}^{\infty }{{g}_{n}}^{2}{f}_{g}({g}_{n}){\rm{d}}{g}_{n},$$

8e $$\langle {G}_{n}\rangle \langle {G}_{m}\rangle ={p}^{2}{\int }_{0}^{\infty }{g}_{n}{f}_{g}({g}_{n}){\rm{d}}{g}_{n}{\int }_{0}^{\infty }{g}_{m}{f}_{g}({g}_{m}){\rm{d}}{g}_{m},$$

*p* is the mean number of domains contained in a cell nucleus, *f*_*z*_(*z*_*n*_) is the probability density of the *z*_*n*_, *f*_*g*_(*g*_*n*_) is the probability density of the DNA amount per domain *g*_*n*_, 〈*G*_*n*_〉 = *p*〈*g*_*n*_〉 is the mean amount of DNA per cell nucleus. For simplicity, we define:

9a $${\alpha }_{n}={A}_{n}\langle {G}_{n}\rangle ,$$

9b $${\beta }_{n}=\frac{{B}_{n}}{p}\langle {{G}_{n}}^{2}\rangle ,$$

9c $${\beta }_{{nm}}=\frac{{B}_{{nm}}}{p}\langle {G}_{n}\rangle \langle {G}_{m}\rangle =\frac{2{B}_{n}}{p}\frac{{c}_{n}}{{c}_{n}+\,{c}_{m}}\langle {G}_{n}\rangle \langle {G}_{m}\rangle \cdot $$

It is assumed that the cells with no LLs (〈*w*_T_〉 = 0) have clonogenic ability. Assuming that the number of LLs per nucleus follows the Poisson distribution, the clonogenic cell survival (*S*) can be expressed by *S* = exp(−〈*w*〉_T_). Thus we obtain the following formula

10 $$\begin{array}{rcl}{\langle w\rangle }_{{\rm{T}}} & = & {\sum }_{n=1}^{N}[({\alpha }_{n}+{\rm{\gamma }}{\beta }_{n})\dot{D}{\Delta }T\,+{\beta }_{n}{(\dot{D}{\Delta }T)}^{2}]\\  &  & +{\rm{2}}{\sum }_{n=1}^{N-1}{\sum }_{m=n+1}^{N}[{\beta }_{{nm}}{e}^{-({\rm{m}}-n)(a+{c}_{n}){\Delta }T}]{(\dot{D}{\Delta }T)}^{2}\\  & = & -\mathrm{ln}\,S.\end{array}$$

**Figure 1:**
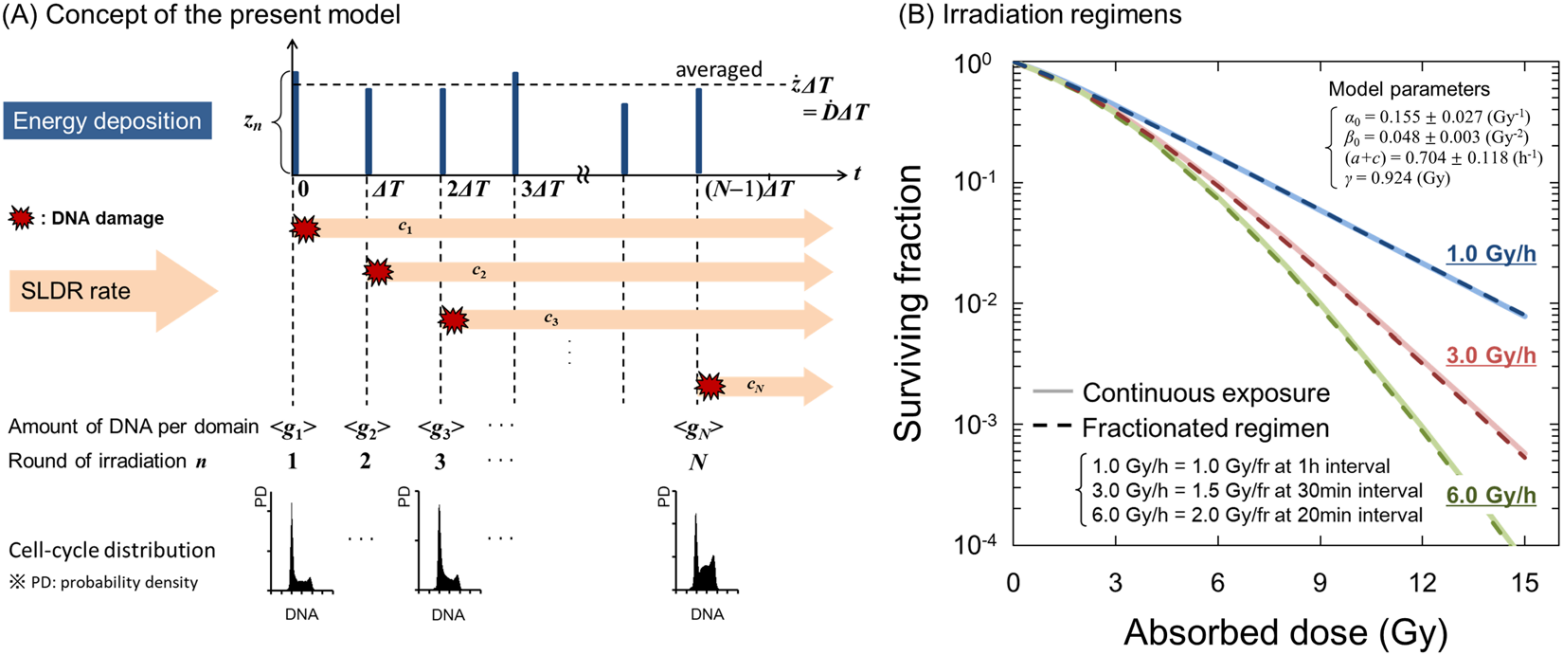
Schematic representation of the present model. (**A**) is the schematic image of the present modelling of cell-killing, and (**B**) illustrates the fractionation regimen equivalent to continuous exposure with a constant dose-rate. The regimen of dose fractionation was determined from the comparison between Eqs (11) and (13). In (**B**), the regimen for 1.0 Gy/h is the same example as described previously^12^.

It should be noted that Eq. (10) represents the relationship between accumulated absorbed dose and surviving fraction of cells (dose-response curve) in consideration of cell cycle (not only the change in amount of DNA, 〈*G*_1_〉, 〈*G*_2_〉, …, 〈*G*_*n*_〉 as previously publised^12^ but also the change in the rate of SLDR, *c*_1_, *c*_2_, …, *c*_*N*_ as newly introduced) (see Fig. 1A).

### Link to the LQ model with or without Lea-Catcheside time factor

The present model can be linked to the LQ model with the classic Lea-Catcheside time factor^19^. Here, we assume a special case that cell condition (amount of DNA and SLDR rate) does not change during irradiation, i.e., 〈*G*_1_〉 = 〈*G*_2_〉 = … = 〈*G*_*N*_〉 = 〈*G*〉 = constant, *c*_1_ = *c*_2_ = … = *c*_*N*_ = *c* = constant, *α*_*n*_ = *α*_0_ = constant and *β*_*n*_ = *β*_*nm*_ = *β*_0_ = constant. Eq. (10) can be expressed by

11 $$\begin{array}{rcl}-\mathrm{ln}\,{S}\, & = & {\sum }_{{\rm{n}}=1}^{N}[({\alpha }_{0}+{\gamma }{\beta }_{0})\dot{D}{\Delta }T+{\beta }_{0}{(\dot{D}{\Delta }T)}^{2}]\\  &  & +2{\sum }_{{\rm{n}}=1}^{N-1}{\sum }_{m=n+1}^{N}[{\beta }_{0}{e}^{-(m-n)(a+{c}_{n}){\Delta }T}]{(\dot{D}{\Delta }T)}^{2}.\end{array}$$

Taking the limit *N* to infinity (hence, *ΔT* → 0), Eq. (11) is approximately expressed by

12 $$\begin{array}{rcl}\mathop{\mathrm{lim}}\limits_{N\to \infty }(\,-\,\mathrm{ln}\,{S})\, & = & \mathop{\mathrm{lim}}\limits_{N\to \infty }{\sum }_{{\rm{n}}=1}^{N}[({\alpha }_{0}+{\gamma }{\beta }_{0})\dot{D}{\Delta }T+{\beta }_{0}{(\dot{D}{\Delta }T)}^{2}]\\  &  & +2\mathop{\mathrm{lim}}\limits_{N\to \infty }{\sum }_{n=1}^{N}{\sum }_{m=n+1}^{N}[{\beta }_{0}{e}^{-(m-n)(a+c){\Delta }T}]{(\dot{D}{\Delta }T)}^{2}.\end{array}$$

Thus we can obtain a simple SF formula considering constant dose rate as

13 $$\begin{array}{rcl}-\mathrm{ln}\,{S} & = & ({\alpha }_{0}+{\gamma }{\beta }_{0})\dot{D}T+\frac{2{\beta }_{0}}{{(a+c)}^{2}{T}^{2}}[(a+c)T+{e}^{-(a+c)T}-\,{\rm{1}}]{(\dot{D}T)}^{2}\\  & = & {\rm{\alpha }}D+\beta {D}^{2}\end{array}\,$$

where

14a $$\alpha ={\alpha }_{0}\,+\gamma {\beta }_{0},$$

14b $$\beta =\frac{2{\beta }_{0}}{{(a+c)}^{2}{T}^{2}}[(a+c)T+{e}^{-(a+c)T}-\,\,1],$$

14c $$D=\dot{D}T.$$

Eq. (13) is the cell-killing model including the Lea-Catcheside time factor including SLDR rate. Further if we consider the acute exposure ($T\to {\rm{0}}$), the following formula of surviving fraction is obtained as

15 $$\begin{array}{rcl}-\mathrm{ln}\,{S} & = & ({\alpha }_{0}+{\gamma }{\beta }_{0})D+{\beta }_{0}{D}^{2}\\  & = & \alpha D+\beta {D}^{2}.\end{array}$$

Thus, the traditional linear-quadratic (LQ) model approximates this model for the case of acute exposure without considering dose-delivery time^2,19^. In this study, in comparison between dose-response curves described by Eq. (11) and by Eq. (13), we planned a fractionated regimen equivalent to the continuous exposure with a constant dose rate (Fig. 1B) as previously described^12^.

## Materials and Methods

### Cell Culture and Irradiation Condition

A mammalian cell line, Chinese Hamster Ovary (CHO)-K1 was obtained from RIKEN Bio Resource Center, Japan (RBC0285). This type of cell line was selected because it does not exhibit HRS^21^. The cells were maintained in Dulbecco’s modified Eagle’s (DMEM, Sigma Life Science) supplemented with 10% fetal bovine serum (FBS, Equitech-Bio Inc.) and 1% penicillin/streptomycin (Sigma Life Science) at 37 °C in humidified 95% air and 5% CO_2_.

To investigate cell responses after a long-term exposure, five days prior to irradiation 4 × 10^5^ cells were seeded onto the cell culture dish with 60 mm diameter (Nippon Genetics) to obtain the cells under plateau phase. In parallel, to quantify the dependence of SLDR rates on cell-cycle distribution, we prepared two cell-cycle distributions for plateau phase and logarithmic growth phase five days and one day after seeding, respectively.

### Irradiation Condition

Standard radiation, 250 kVp X-rays (Stabilipan, Siemens, Concord, CA), was used to irradiate the cultured cells. The dose rate in air at the surface of cell culture was measured by using Farmer-type ionizing radiation chamber (model NE2581, Nuclear Enterprises Ltd) and was converted to the dose rate in water (4.31 Gy/min) according to the dose protocol TRS277^31^. From the comparison between Eqs (11) and (13), the practical fractionation regimens equivalent to the average dose rates of 3.0 Gy/h and 6.0 Gy/h were newly determined at a visual level (the *R*^2^ value is found to be 0.999), which were 1.5 Gy per fraction at 30 min interval for 3.0 Gy/h and 2.0 Gy at 20 min interval for 6.0 Gy/h (Fig. 1B).

### Flow-Cytometric Analysis of Cell-Cycle Distribution

For each dose-rate exposure, 1 × 10^6^ cells were harvested at 0, 2, 4, 6, 8, 10, 12 h after the start of irradiation and fixed with 70% ethanol, and then kept at 4 °C for at least 2 h. After a centrifugation, the cells were re-suspended in 1 ml phosphate-buffered saline (PBS) (*−*). After a centrifugation again, the DNA was stained with 0.5 ml FxCycle^TM^ propidium iodide (PI)/RNase staining solution (Life Technologies) including 0.2% v/v triton X for 15 min in the dark at room temperature. Cell-cycle distribution was then obtained by using the Attune acoustic focusing flow cytometer (Applied Biosystems by Life Technologies TM).

The fluorescence intensity emitted from the DNA in a nucleus was normalized by that from the DNA contained in a cell in G_0_/G_1_ phase. The cell-cycle distribution (fractions of the cells in G_0_/G_1_, S and G_2_/M) was then obtained from the DNA profile. All sets of cell-cycle study were performed three times. By using the Tukey-Kramer method^32^, we evaluated if there is significant difference in the change of cell-cycle distribution from the control group (before irradiation at *t* = 0 (h)).

### Clonogenic Survival Assay

After exposure to the regimen equivalent to 3.0 and 6.0 Gy/h, irradiated cells were trypsinized immediately and the appropriate number of cells was reseeded into a cell culture dish with 60 mm diameter (Nippon Genetics). Culture medium was exchanged every two days and the cells were cultured for 10–14 days. The colonies were fixed with methanol and were stained with 2% Giemsa solution (Kanto Chemical Co. Inc.) Survival fraction was obtained from the ratio of colony number of irradiated cells to that of non-irradiated cells (control cells). The plating efficiency for control cells was 38.8 ± 9.2% (mean ± standard deviation), which was given by 27 dishes (the assay was performed three times for each dose-rate, and three dishes were used in one assay).

### Determination of SLDR Rate from a Split-Dose Cell Recovery Curve

The constant rate of SLDR was obtained from cell recovery curve of cell survival in a split-dose experiment. Let us consider a case of exposing a cell population to equal acute doses with *D*_1_ (=*<z*_1_>) (Gy) and *D*_2_ (=*<z*_2_>) (Gy) at the interval of *τ* (=*ΔT*) (h). The surviving fraction for a split-dose exposure is given by

16 $$-\,\mathrm{ln}\,S\,(\tau )={\sum }_{n=1}^{2}[({\alpha }_{0}+\gamma {\beta }_{0}){D}_{n}+{\beta }_{0}{{D}_{n}}^{2}]+{\rm{2}}{\beta }_{0}{e}^{-(a+c)\tau }{D}_{1}{D}_{2}.$$

The SLDR rate can be deduced by using the surviving fractions taking the limits of period of exposure interval (${\tau }\to {\rm{0}},\,\,{\tau }\to \infty$). Based on Eq. (16), *S*(0) and *S*($\infty$) can be given by

17 $$-\mathrm{ln}\,S(0)={\sum }_{n=1}^{2}[({\alpha }_{0}+\gamma {\beta }_{0}){D}_{n}+{\beta }_{0}{{D}_{n}}^{2}]+{\rm{2}}{\beta }_{0}{D}_{1}{D}_{2},$$

18 $$-\mathrm{ln}\,S(\infty )={\sum }_{n=1}^{2}[({\alpha }_{0}+\gamma {\beta }_{0}){D}_{n}+{\beta }_{0}{{D}_{n}}^{2}].$$

On one hand, subtracting Eq. (18) from Eq. (17) gives

19 $$-\,\mathrm{ln}\,S(0)-(-\,\mathrm{ln}\,S(\infty ))={\rm{2}}{\beta }_{0}{D}_{1}{D}_{2}.$$

On the other hand, taking the derivative of Eq. (16) and taking the limit of d*S*/d*τ* as *τ* tends to 0, we have

20 $$\mathop{\mathrm{lim}}\limits_{\tau \to 0}\frac{1}{S}\frac{{\rm{d}}S}{{\rm{d}}\tau }={\rm{2}}{\beta }_{0}{D}_{1}{D}_{2}(a+c){\rm{.}}$$

Thus we can deduce the cell-specific parameter of SLDR rate by using the following equation,

21 $$(a+c)=\frac{\mathop{\mathrm{lim}}\limits_{\tau \to 0}\frac{1}{S}\frac{dS}{d\tau }\,}{\mathrm{ln}\,\frac{S(\infty )}{S(0)}}.$$

Cell recovery curves for a split-dose experiment are always influenced by re-distribution and cell proliferation^17,18^. Based on the previous reports to deduce (*a* + *c*) value^17,18^, to avoid the influences of re-distribution and repopulation, the initial slope d*S*/d*τ* and *S*($\infty$) were determined from experimental surviving fraction by taking the gradient from 0 to 1 h, and maximum survival was taken at the 2 h time interval. Because the value of *a* is a few percent of *c*^17,20^, thus the rate of SLDR can be approximated by *c* value. Whilst the SLDR rate for plateau phase of CHO-K1 was taken from our previous report^12^, that for logarithmic growth phase was deduced by Eq. (21) and a cell recovery curve reported in the literature^33^.

### Change of DNA Amount and SLDR Rate During Irradiation

Experimentally determined changes of relative DNA amount per nucleus during irradiation 〈*G*_*n*_〉/〈*G*_1_〉 were input into Eq. (10) as previously described^12^. Additionally, in this study we assumed that SLDR rate represented by *c*_*n*_ (h^−1^) changes during irradiation depending on fraction of cells in S phase, which has a high repair efficiency^34^ leading to lower radio-sensitivity^14,35^. We estimated the differential rate of SLDR, and then deduced the change of SLDR rate per fraction of cells in S phase during exposure from experimental cell-cycle distributions using the following equation

22 $${c}_{n}={c}_{{\rm{0}}}+\frac{{\rm{d}}c}{{\rm{d}}{N}_{{\rm{S}}}}{\rm{\Delta }}{N}_{S}(t),$$

where *t* is the time after the start of irradiation (h), *c*_0_ is the SLDR rate at *t* = 0, d*c*/d*N*_*S*_ is the differential rate of SLDR per fraction of cells in S phase, and *N*_*S*_ is the fraction of cells in S phase. In this study, d*c*/d*N*_*S*_ was determined from the subtractions of SLDR and fraction of cells in S phase for plateau and logarithmic growth phases, and the change of cell fraction in S phase Δ*N*_*S*_ was obtained from cell-cycle study during fractionated exposures.

### Comparison Between Model and Measured Cell Survival

To investigate the influence of change of repair rate associated with cell-cycle distribution on cell survival curve, we compared the dose-response curves estimated by Eq. (10) with measured clonogenic cell survival data. We used model parameters for the CHO-K1 cell line after exposure with 250 kVp X-rays which have been already published^12^. The values of the parameters are summarized in Table 1. In the set of parameters, DNA contents represented by 〈*G*〉 and 〈*G*^2^〉 were obtained within the performed cell-cycle experiment, and the set of physical parameters (*γ*, *y*_*D*_, *r*_d_, *ρ*) was taken from the previous reports^12^. Here, we assumed that the constant rates of *a* (h^−1^) and *b*_d_ (h^−1^) are cell-specific parameters independent of the cell-cycle distribution for simplicity, and the cell survival curve was calculated considering the change of mean DNA amount per nucleus and SLDR rate during the exposure at various dose-rates.

**Table 1:** Model parameters for two cell conditions in CHO-K1 cell line.

Model Parameters	Cell Condition	
	Plateau Phase	Logarithmic Growth Phase
*α*_1_ = *A*_1_〈*G*_1_〉(=*α*_0_) (Gy^−1^)	0.155 ± 0.027	0.075 ± 0.025
*β*_1_ = *B*_1_〈*G*_1_^2^〉 (=*β*_0_) (Gy^−2^)	0.048 ± 0.003	0.028 ± 0.008
$(a+{c}_{1})\cong {c}_{1}(=c)\,({{\rm{h}}}^{-1})$	0.704 ± 0.118	1.782 ± 0.441
Relative 〈*G*_1_〉 (=〈*G*〉)	1.000 ± 0.003	1.227 ± 0.009
Relative 〈*G*_1_^2^〉 (=〈*G*^2^〉)	1.000 ± 0.007	1.457 ± 0.050
*ρ* (g · cm^−3^)	1.000 (water)	1.000 (water)
*r*_d_ (μm)	0.500	0.500
$\gamma ={y}_{D}/({\rm{\rho }}{\rm{\pi }}{r}_{{\rm{d}}}^{{\rm{2}}})\,({\rm{Gy}})$	0.924 (250 kVp)	0.924 (250 kVp)
**Each Phase**	**Cell Cycle distribution [%]**	
	**Plateau Phase**	**Logarithmic Growth Phase**
G_0_/G_1_	72.7 ± 6.6	37.6 ± 3.8
S	14.9 ± 2.3	52.4 ± 4.5
G_2_/M	12.4 ± 3.9	11.4 ± 0.7

The set of values for plateau phase was obtained from our previous report^12^. In these parameters, *a* is a few percentage of *c*^17,20^. Relative mean DNA amount per nucleus was normalized by that for plateau phase. The set of parameters for logarithmic growth phase was converted from that for plateau phase (SD was deduced by error propagation). In the parameters, (*a* + *c*) value for logarithmic phase was deduced from an experimental cell recovery data^33^, and DNA amount represented by 〈*G*〉 and 〈*G*^2^〉 was obtained within the performed cell-cycle experiment. The set of physical parameters (*γ*, *y*_*D*_, *r*_d_, *ρ*) was taken from the previous reports^12^.

To test the assumption of fixed *a* and *b*_d_ values, we compared the estimated survival curve with experimental one for two cell conditions of plateau and logarithmic growth phases. Whilst the set of parameters (*α*_0_, *β*_0_, *a* + *c*) for plateau phase is summarized in the left side of Table 1, the set for logarithmic growth phase is given by the ratios of DNA amount measured in this work and SLDR rate deduced by Eq. (21).

The fit quality of the model used in this study was evaluated from reduced chi-square value expressed by

23 $${\chi }^{2}=\frac{1}{n}{\sum }_{i=1}^{n}\frac{{({S}_{{\rm{i}}\exp }-{S}_{i{\rm{model}}})}^{2}}{{\sigma }_{i\,\exp }^{2}},$$

where *S*_exp_ is measured cell survival, *S*_model_ is cell survival estimated by the present model, *σ*_exp_ is the standard deviation of measured cell survival.

### Mean Inactivation Doses

To investigate survival curves of the CHO-K1 cells, we further used the concept of the mean inactivation dose $\bar{D}$^36^, which is recommended by ICRU Report 30^37^. In this quantity, dose-response curve is treated as a probability distribution of cell killing with absorbed dose. Considering the survival probability *S*(*D*) as an integral probability distribution, the mean dose necessary to inactivate cells (so-called mean inactivation dose) $\bar{D}$ is given as,

24 $$\bar{D}={\int }_{0}^{\infty }S(D){\rm{d}}D\,{\rm{.}}$$

The $\bar{D}$ values for various dose-rates of 18.6–60.0 Gy/h were calculated for experimental survival data, model prediction at constant SLDR of *c* $\cong$ (*a* + *c*) = 0.704 (h^−1^) based on Eq. (13), and the prediction considering changes of mean DNA contents per nucleus and S-phase dependent SLDR rate based on Eq. (10). The $\bar{D}$ values predicted by the model were compared with the experimental data by using *R*^2^ value given by

25 $${R}^{2}={\rm{1}}-\frac{{\sum }_{i=1}^{n}{({ex}{{p}}_{i}-{ex}{{p}}_{i})}^{{\rm{2}}}}{{\sum }_{i=1}^{n}{({ex}{{p}}_{{i}}-\langle {\exp }\rangle )}^{2}},$$

where *exp* and *est* represent the experimental value and the estimated value by the model, respectively, and *n* is the number of data.

## Results

### Change in Cell-Cycle Distribution during Exposure for Various Dose Rates

**Figure 2:**
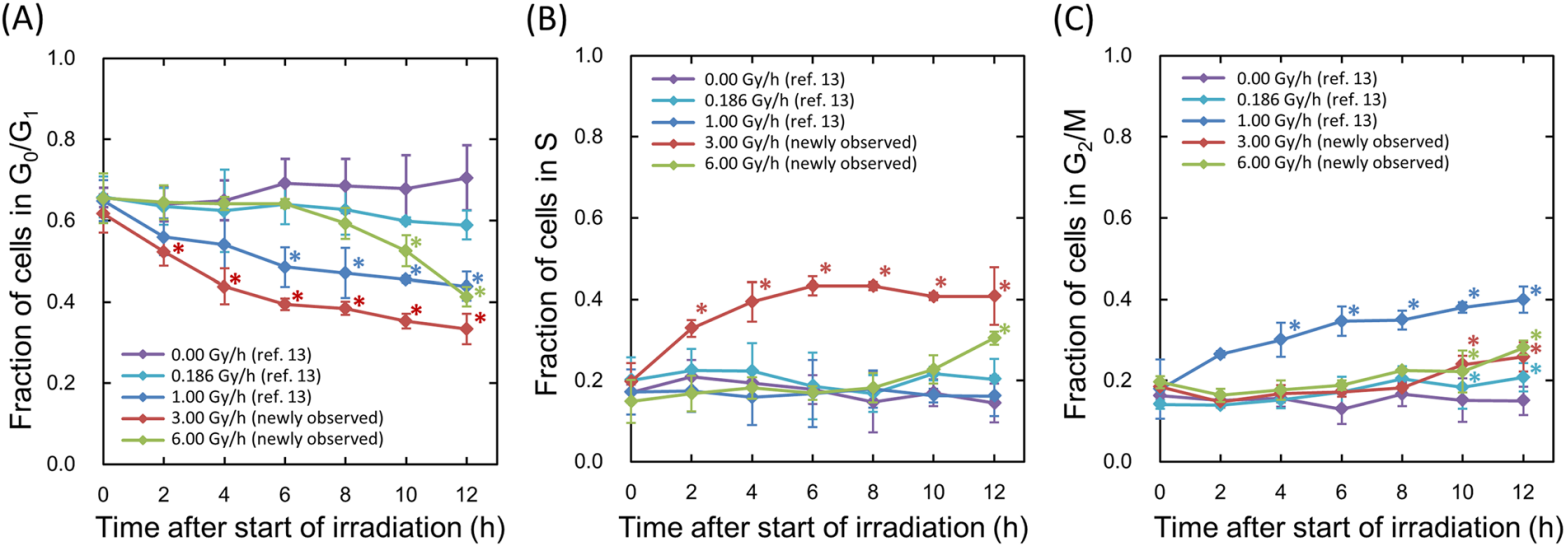
Cell-cycle distribution during exposure at various dose-rates. (**A**) is for the change of cell faction in G_0_/G_1_ phase, (**B**) is for that in S phase and (**C**) is for that in G_2_/M phase. The data for 3.0 and 6.0 Gy/h were newly measured in the present flow-cytometric analysis and the other sets of data were taken from our previous report^12^. The symbol * represents P < 0.05 significant change compared with the data at just start of irradiation (0 h). The error bar represents the standard deviation deduced from three independent experiments. In addition, we confirmed there is no significant difference of cell-cycle distribution at the starting time of irradiation among control (0.00 Gy/h), 0.186 Gy/h, 1.0 Gy/h, 3.0 Gy/h and 6.0 Gy/h by using the Tukey-Kramer method.

Two fractionated regimens equivalent to the continuous exposures with 3.0 and 6.0 Gy/h were used to investigate DREs on cell-cycle distribution during exposure. Figure 2 shows the change in cell-cycle distribution during the exposure for various dose-rates, in which data at 3.0 Gy/h and 6.0 Gy/h were newly measured in this study. The data at 0.0 Gy/h, 0.186 Gy/h and 1.0 Gy/h were obtained from our previous investigation^12^.

During the exposure with lower dose-rates such as 1.0 Gy/h and 0.186 Gy/h, the fraction of the cells in G_2_/M phase gradually and slightly increased with increasing fractions. During the exposure with 3.0 Gy/h, significant increases of cell fraction in S phase were observed up to 6 h after the start of fractionated irradiation, whilst the subtle accumulation of the cells in G_2_/M phase was observed. During the exposure with the highest dose rate, 6.0 Gy/h, there is no change in cell-cycle distribution until 8 h after the start of irradiation. However, at 10 h after the start of irradiation, significant increases of cell fraction in S phase and G_2_/M phase were observed.

### SLDR Rate during Exposure and Prediction of Cell Survival

**Figure 3:**
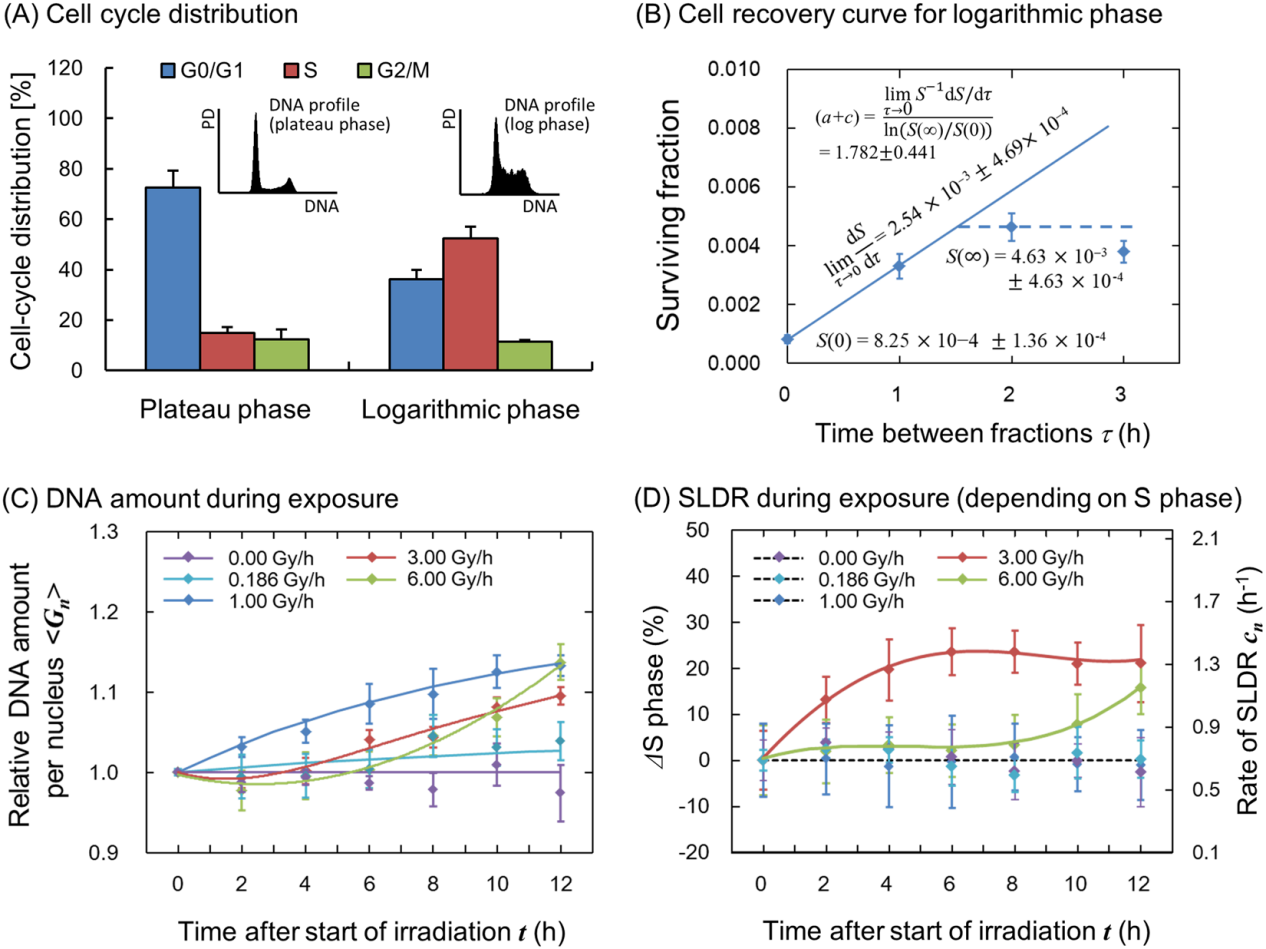
Cell condition (DNA profile and SLDR rate) input into the present model. (**A**) shows DNA profile and cell-cycle distribution for plateau and logarithmic growth phase in CHO-K1 cell line, (**B**) shows procedure to deduce the rate of SLDR for logarithmic growth phase, (**C**) and (**D**) show the change of average DNA amount per nucleus and SLDR rate during protracted exposure, respectively, for various dose-rates. In Fig. 3B, a split-dose cell recovery data (5 Gy + 10 Gy) was taken from ref.^33^ and we deduced (*a* + *c*) value based on Eq. (21) from d*S*/dτ, *S*(0) and *S*($\infty$). Here the data for 0.0, 0.186 and 1.0 Gy/h were taken from our previous investigation^12^. In Fig. 3C,D, whilst the symbols represent measured mean DNA contents and variation of S-phase fraction, the lines are the interpolated curve by spline. The constant rates for the exposure to 0.0, 0.186 and 1.0 Gy/h of SLDR were adopted because there is no significant change of S-phase fraction.

To investigate the influence of the change in cell-cycle distribution on cell survival, we obtained the change of mean DNA amount per nucleus and the SLDR rate during exposure from flow-cytometric analysis of DNA profiles. In the upper panels of Fig. 3, the DNA profile for plateau phase and for logarithmic growth phase (Fig. 3A) and the procedure to deduce the SLDR rate for logarithmic growth phase from Eq. (21) with a split-dose cell recovery^33^ (Fig. 3B) are presented. The details of cell-cycle distribution for both phases and SLDR rates *c*_1_ were also listed in Table 1. The SLDR rate for logarithmic growth phase (1.782 ± 0.441 h^−1^) is in good agreement with that of the previous report (1.72 (1.27–2.59) h^−1^) by Hawkins^17^. Based on the cell-cycle study (Fig. 2) and the rate of SLDR via cell-killing model (Fig. 3B), we obtained the change of mean DNA amount per nucleus (Fig. 3C) and estimated the S-phase dependent SLDR rate during exposure according to Eq. (22), as shown in Fig. 3D. From the experimental data about fraction of cells in S phase and SLDR rate for plateau and logarithmic growth phases (Table 1), we deduced the value of d*c*/d*N*_*S*_ = 0.0287 ± 0.0128 (h^−1^/%).

**Figure 4:**
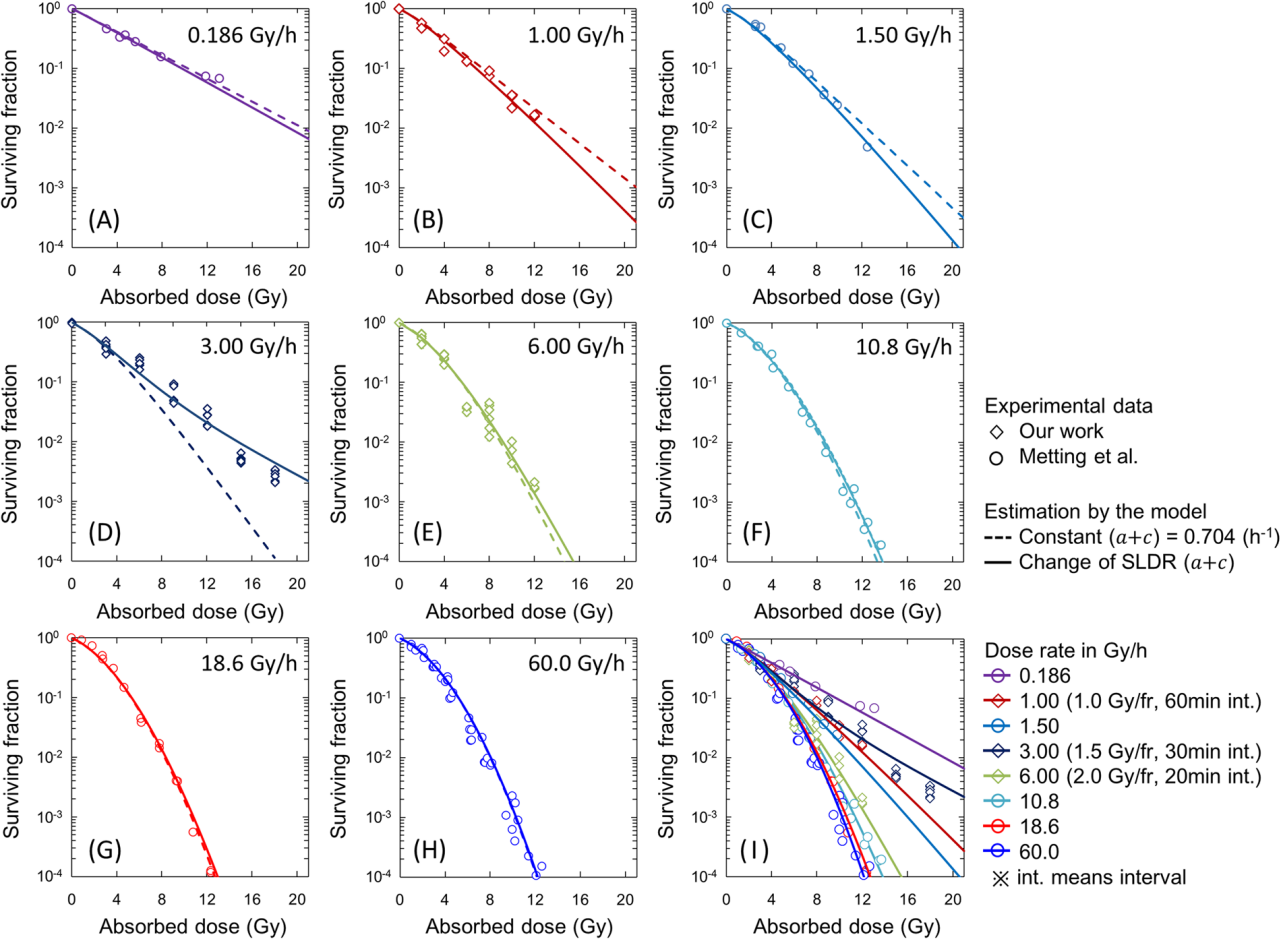
Comparison between the clonogenic survival data and the model prediction. Whilst the symbols denote the survival data by our work and reference data, dotted line and solid line represent the model estimations with a constant rate of (*a* + *c*)  =  0.704 (h^−1^) and with changing rate of *c* based on Fig. 3D, respectively. The data for 3.0 and 6.0 Gy/h were newly observed in this paper. The fit qualities of the model for 1.0 Gy/h, 3.0 Gy/h and 6.0 Gy/h are summarized in Table 2.

Figure 4 shows the comparison between the cell survival curve described by the present model (IMK model) and the clonogenic survival data for various dose-rates of 0.186–60.0 Gy/h. This includes both newly obtained dose-response curves after the exposure with 3.0 and 6.0 Gy/h (Fig. 4D,E) and re-analysed curves in comparison with reference data (Fig. 4A–C and F–H)^38^. In Fig. 4, dotted lines and solid lines represent respectively the model prediction according to Eq. (13) with a constant rate of (*a* + *c*)  =  0.704 (h^−1^) and Eq. (10) with the experimental-based variable SLDR rate during exposure as well as DNA amount changing shown in Fig. 3C,D, whilst symbols denote the experimental cell survival including reference data^38^. In Fig. 4D for 3.0 Gy/h, unexpected radio-resistance (increase of cell survival) was observed compared to the curve predicted by Eq. (13). This is attributable to both the change of SLDR rate and DNA amount per nucleus during exposure (Fig. 3C,D). The cell survival curves (in Fig. 4B,D and E) described by the IMK model for 1.0, 3.0 and 6.0 Gy/h with the both factors were in better agreement with experimental data (Table 2). Applying the time course of DNA contents and SLDR rate under 6.0 Gy/h into the model prediction of cell survival for a higher dose rate of 10.8 Gy/h, the surviving fraction estimated by the IMK model is slightly higher than that by the previous model (Fig. 4F). Figure 4G,H represent the cases in higher dose rates (18.6 and 60.0 Gy/h), where the dose-delivery time is relatively short. We applied the cell-cycle kinetics under higher dose-rate of 6.0 Gy/h to predict the dose-response curve for 18.6 and 60.0 Gy/h.

**Table 2:** Statistical evaluation of fit quality of the present model.

Dose Rate (DR)	*χ*^2^ value	
	Constant SLDR (*a* + *c*) = 0.704	DNA amount & changing SLDR
1.00 Gy/h	9.27 × 10^−1^	5.33 × 10^−1^
3.00 Gy/h	1.09 × 10^1^	3.41 × 10^0^
6.00 Gy/h	1.18 × 10^0^	8.28 × 10^−1^
Sum *χ*^2^ value	1.30 × 10^1^	4.77 × 10^0^

The *χ*^2^ value was calculated by using Eq. (23).

Figure 4I shows the collection of all of the parts of dose-response curve estimated by the present model (following Eq. (10) and change of cell conditions given by Fig. 3C,D) in comparison with the experimental data. The cell survival increases as the dose-rate decreases by virtue of SLDR during dose-delivery time. However, focusing on the dose-rate range of 1.0–3.0 Gy/h, the inverse dose-rate effects (IDREs) can be observed, in which a radio-resistance resulted from cell accumulation in S phase for 3.0 Gy/h whilst a higher radio-sensitivity induced by cell accumulation in G_2_/M phase for around 1.0 Gy. These results suggest that the cell-cycle dynamics during exposure may modulate the cell survival curve, whilst it is shown that the cell-killing model with traditional Lea-Catcheside time factor (Eq. (13)) is a special case for no cell-cycle change during exposure.

### Evaluation of Dose-Rate Effects on Mean Inactivation Dose

**Figure 5:**
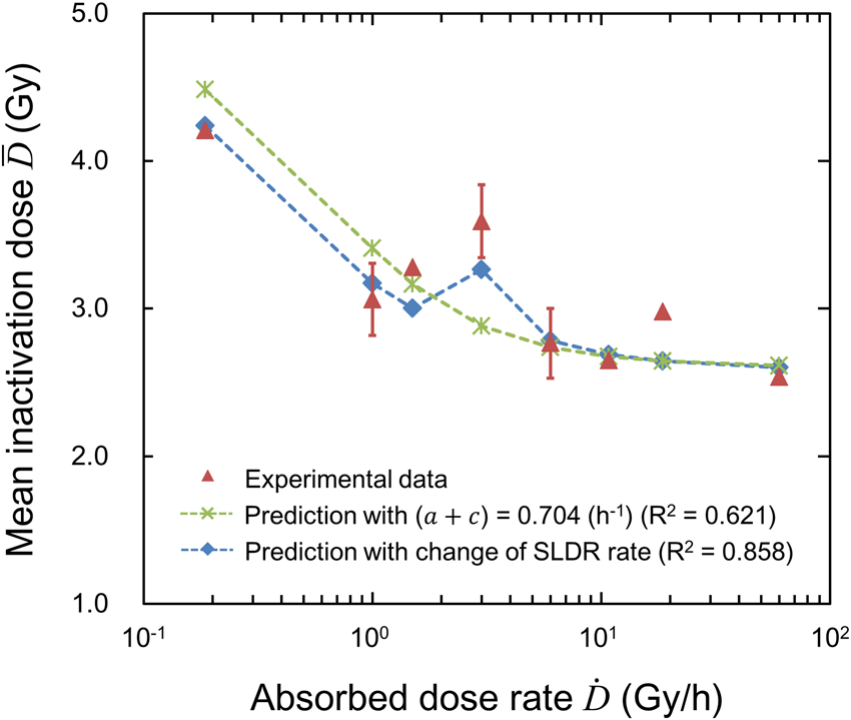
Mean inactivation dose $\bar{D}$ for evaluating the DREs. Red symbols denote the experimental $\bar{D}$, green line (with symbols) is the $\bar{D}$ calculated by using the cell-killing model formula Eq. (13) with constant (*a* + *c*) value of 0.704 (h^−1^), and blue line (with symbols) is the $\bar{D}$ calculated by using the model (Eq. (10)) with variable repair rate, *c*, during the exposure. It is noted that there is concave characteristics in dose-rate range of around 1.5–3.0 Gy/h. The $\bar{D}$ and *R*^2^ values were calculated by using Eqs (24) and (25), respectively.

The DREs on cell survival shown in Fig. 4 were next evaluated by means of the mean inactivation dose $\bar{D}$^35^, which is recommended by ICRU Report 30^37^. Figure 5 shows the relationship between the absorbed dose rate in Gy/h and mean inactivation dose $\bar{D}$, in which experimental $\bar{D}$ was represented as red symbol. In this study, we predicted the DREs by using two SLDR approach, one for a constant (*a* + *c*) value of 0.704 (h^−1^) and the other for the variable (*a* + *c*) values during irradiation shown in Fig. 3D. In Fig. 5, whilst the $\bar{D}$ value predicted with (*a* + *c*)  =  0.704 (h^−1^) becomes higher as the dose-rate is lower (green symbols and dotted line), the predicted $\bar{D}$ with variable SLDR rate (blue symbols and dotted line) agrees better with the experimental $\bar{D}$. In comparison between the experimental and model predicted values, it was shown that the IMK model with variable (*a* + *c*) leads to a peak of resistance at dose-rates around 1.5–3.0 Gy/h in agreement with the experimental result. The experimental $\bar{D}$ value at 10.8 Gy/h is higher than that by the model, suggesting a higher radio-resistance. However, the experimental data is calculated from just one data set result taken from ref.^38^. Regarding the about 5% inherent uncertainties of $\bar{D}$ value^36^ and the possibility of experimental outliers, we cannot clearly judge if there is a reversal in radio-sensitivity at dose-rates around 10.8 Gy/h.

### Testing the Assumption of Cell-Specific Model Parameters Independent of the Cell Cycle

**Figure 6:**
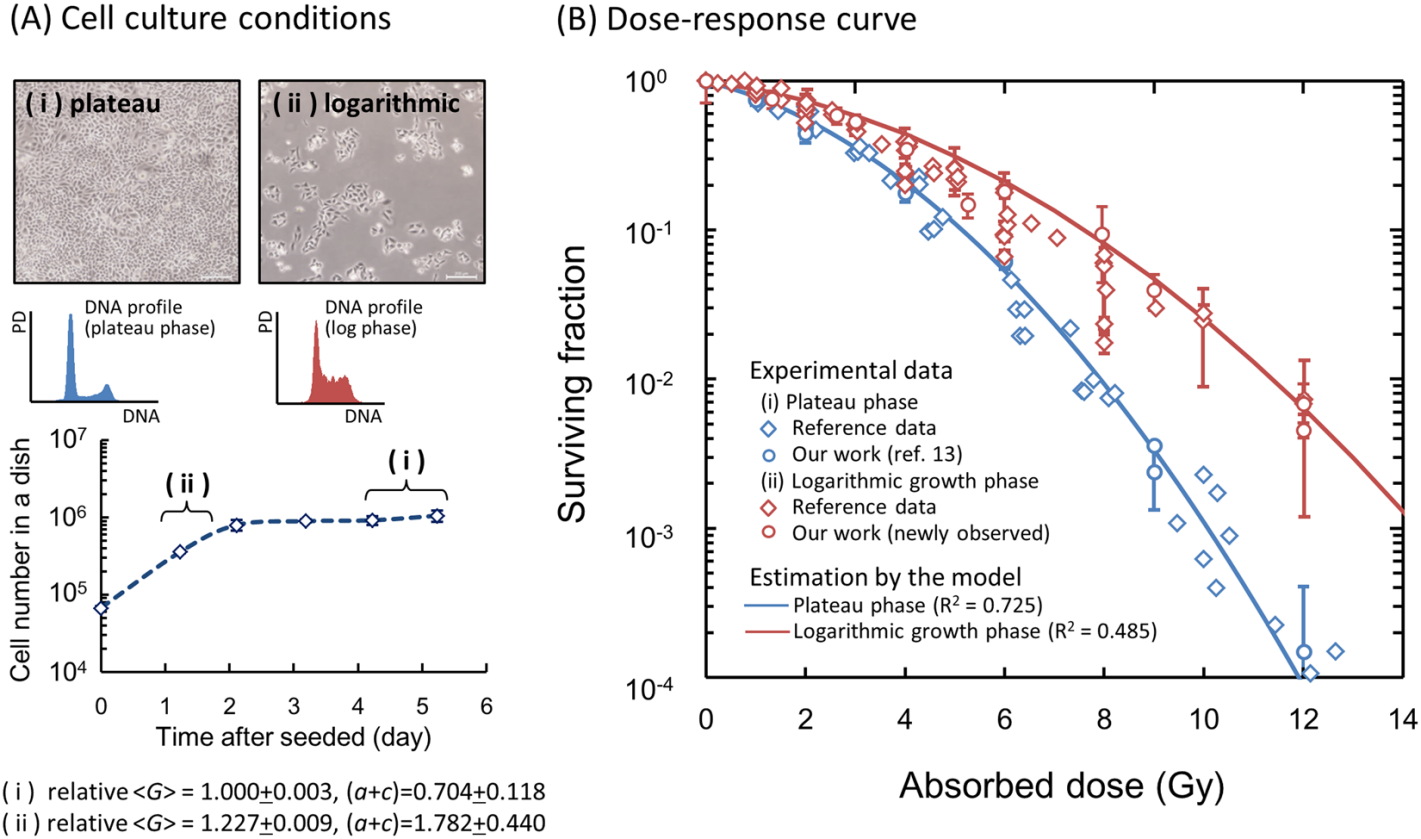
Dose-response curves for different cell culture conditions. (**A**) Upper panels represent microscopic images and DNA profiles for (i) plateau and (ii) logarithmic growth phases, respectively. Bottom figure is cell growth curve of CHO-K1 cell line to make the two cell conditions. (**B**) Dose-response curves were predicted by considering DNA contents per nucleus and the rate of SLDR for the two different phases. The set of parameters for logarithmic growth phase listed in Table 1 was deduced from that for plateau phase with the cell condition (DNA content and SLDR rate). The estimated curves were compared with experimental data after irradiation with 250 kVp X-rays^2,38–44^ to check the assumption in this study.

Testing the Assumption of Cell-Specific Model Parameters Independent of the Cell Cycle. Here we assumed that the constant rates of *a* (h^−1^) and *b*_d_ (h^−1^) are cell-specific parameters independent of the cell-cycle distribution. To check if this assumption is correct or not, we further compared Eq. (15) with measured cell survival after acute exposure under two cell phases, plateau phase and logarithmic growth phase (Fig. 6A). The set of parameters (*α*_0_, *β*_0_) for plateau phase was converted to those in logarithmic growth phase by using the ratios of DNA amount and SLDR rate, which is listed in Table 1. In Fig. 6B, we also compared the cell surviving fractions for both phases^2,38–44^ with the estimation by using the converted model parameters for logarithmic growth phase. In this comparison, the difference between dose-response curves in plateau phase and logarithmic growth phase is explainable by taking account of the mean DNA amount per nucleus and SLDR rate, which validates partly the interpretation that the values of *a* (h^−1^) and *b*_d_ (h^−1^) don’t depend on the cell condition.

## Discussion

CHO-K1 cells show the following responses: (i) accumulation of cells in G_2_ during exposure with 1.0 Gy/h^12^, (ii) delay of DNA synthesis and accumulation of the cells in S and G_2_ during exposure with 3.0 Gy/h and (iii) no significant change of cell-cycle distribution until 8 h after the start of exposure to 6.0 Gy/h (Fig. 2). The increase of cells in S phase during the exposure to 3.0 Gy/h might be attributed to the DNA damage response in S phase checkpoint^45^. In contrast, according to previous investigations^46^, the threshold dose for blocking cell cycle progression at the G_1_/S checkpoint is considerably higher than G_2_/M checkpoint. For the case of CHO cells, Lee *et al*. reported a p53-independent damage-sensing checkpoint which operates to prevent late G_1_ or early S-phase^47^. In this regard, we interpreted that whilst the G_1_/S checkpoint system was not activated under exposure with 3.0 Gy/h, it was activated under exposure with 6.0 Gy/h (Fig. 2A).

From the measured cell-cycle distribution (Fig. 2), we estimated S-phase dependent SLDR rates (Fig. 3D) and subsequently described cell survival curves in comparison with the MK model with a constant SLDR rate of (*a* + *c*)  =  0.704 (h^−1^) (dotted line in Fig. 4) and also with a changing rate of SLDR (solid line in Fig. 4). From the statistical evaluation by using the model (Table 2), the changing rate of repair *c* plays an important role for describing not only the DREs on cell survival curve with reduction of chi-square value (Fig. 4 and Table 2) but also mean inactivation dose with reasonable *R*^2^ value (Fig. 5). This suggests that the magnitude of DNA damage might trigger the series of repair proteins activation, depending on dose rate^48^. Here, the misrepair rates, *a* (h^−1^) and *b*_d_ (h^−1^) were assumed to be constant cell-specific parameters not depending on cell condition. On this basis, we converted the set of parameters for plateau phase to that for logarithmic growth phase (Table 1), to reproduce the surviving fraction under the both phases in the methodology of the IMK model (Fig. 6B). According to the previous model assessment by Hawkins, the value of (*a* + *c*) is mainly composed of non-homologous end joining (NHEJ)^20^. However, it is known that Homologous Recombination (HR), a more accurate repair process, becomes more important in S phase, which may contribute to the observed increased resistance in S phase. Traditionally, there are two types of definition for repair kinetics, i.e., SLDR for dose fractionation and potentially lethal damage repair (PLDR) for treatment such as hypertonic saline. Several attempts have been made to understand whether or not SLDR and PLDR are the same^49^. To this subject, the present model study (Figs 4 and 6B) may support that SLDR and PLDR are similar to each other.

The model proposed in this study follows the linear-quadratic (LQ) formalism, which is convenient for calculating *α/β* and biological effective dose (BED)^50^ considering the Lea-Catcheside time factor^19,51^. The modelling approach for predicting cell-cycle dependent survival of V79 cells was also presented in a similar manner to that with a small set of parameters by Hufnagl *et al*.^53^. In comparison to their model, the present IMK model enables us to describe cell-cycle dependent dose-response curve (Fig. 4) for not only acute exposure but also long-term exposure (Fig. 6B). However, the DNA damage repair kinetics is complex and is generally quantified by two exponential components for rejoining of the broken ends of DNA^52^. If the complex repair kinetics is essential, more detailed mechanistic modelling such as the computational modelling by McMahon *et al*.^54,55^ may be more suitable to understand the underlying radiation biology. As for clarifying the underlying mechanism of damage repair system, further investigation about the relation between SLDR and repairs function (NHEJ, HR, etc.) is necessary in various cell lines.

The model and data exhibit IDREs in the dose-rate range of 1.0–3.0 Gy/h, attributable to increases in SLDR during irradiation. According to the previous investigation about dependence of cell phase on cell killing^14^, S phase (including late S phase) is the most radio-resistant. This tendency was found in the comparison between the experiment and the model in Fig. 4D–F. In addition, the accumulation of cells in the relatively more radio-sensitive phase of G_2_/M^12,14,56^ contributes to the modulation of cell survival curve, leading to the reversal of DREs as shown in Fig. 4I. Supported by the dose-response curve in Fig. 4I, the mean inactivation dose in Fig. 5 indicates the existence of IDREs as well. Considering these results, the combination of the accumulations of cells in S phase and G_2_/M phase is possibly responsible for IDREs.

IDREs on cell killing have been previously observed in dose-rate range of below 1.0 Gy/h^5,57^, and other reports have shown higher mutant frequencies at dose-rates lower than 0.1–1 cGy/min^58^. This previously observed dose-rate range is different from that we observed in this study. Other potential mechanisms, such as cumulative low-dose HRS after fractionated exposures has a possibility to induce this reversal in radio-sensitivity^57–60^, however the CHO-K1 cell line does not exhibit this behaviour^21^. From the present study and the previous reports, it is likely that the dose-rate range for IDREs related to cell-cycle effects is higher than that related with low-dose HRS. Further model development for the cumulative low-dose HRS based on more detailed mechanistic evidence about the time course of the HRS is necessary.

In summary, we investigated the radio-sensitivity after the protracted exposure for various dose rates. Focusing on cell-cycle distribution, the experimental results suggested that the CHO-K1 cells show following responses: (1) an accumulation of the cells in G_2_ during exposure with lower dose rate (e.g., 1.0 Gy/h), (2) the delay of DNA synthesis and an accumulation of the cells in S/G_2_ during the exposure with intermediate dose rates (e.g., 3.0 Gy/h), and (3) the blocks of cell cycle progressing in whole checkpoints (G_1_/S and G_2_/M checkpoints) and the delay of DNA synthesis during the exposure with higher dose (e.g., 6.0 Gy/h). A greater radio-resistance after the exposure with 3.0 Gy/h was observed and this tendency was interpreted as increases in SLDR rate associated with the fraction of cells in S phase. Taking account of both higher radio-sensitivity under 1.0 Gy/h exposure and the radio-resistance after exposure to 3.0 Gy/h, the changes in cell-cycle distribution during exposure modulate the cell survival curve and are possibly responsible for IDREs. This study would contribute to a quantitative understanding of radio-sensitivity after long-term exposure to ionizing radiation as well as general characteristics of DNA repair and dose response, which can be of help to radiation protection and radiation therapy.

## References

[1] van der Kogel, A. J. The dose-rate effect. In: Joiner, M., van, der, Kogel, A. J. (eds). Basic Clinical Radiobiology. London, Hodder Arnold, 158–168 (2009).

[2] Matsuya Y, Tsutsumi K, Sasaki K, Date H (2015). Evaluation of the cell survival curve under radiation exposure based on the kinetics of lesions in relation to dose-delivery time. J. Radiat. Res..

[3] Amundson SA, Chen DJ (1996). Inverse dose-rate effect for mutation induction by γ-rays in human lymphoblasts. Int. J. Radiat. Biol..

[4] Stevens DL, Bradley S, Goodhead DT, Hill M (2014). The influence of dose rate on the induction of chromosome aberrations and gene mutation after exposure of plateau phase V79-4 cells with high-LET alpha particles. Radiat. Res..

[5] Mitchell CR, Folkard M, Joiner MC (2002). Effects of exposure to low- dose-rate ^60^Co gamma rays on human tumor cells *in vitro*. Radiat. Res..

[6] Marples B, Wouters BG, Collis SJ, Chalmers AJ, Joiner MC (2004). Low-Dose Hyper-radiosensitivity: A Consequence of Ineffective Cell Cycle Arrest of Radiation-Damaged G2-Phase Cells. Radiat. Res..

[7] Joiner MC, Marples B, Lambin P, Short SC, Turesson I (2001). Low-dose hypersensitivity: current status and possible mechanisms. Int. J. Radiat. Oncol. Biol. Phys..

[8] Prise KM, O’Sullivan JM (2009). Radiation-induced bystander signal- ling in cancer therapy. Nat. Rev. Canc..

[9] McMahon SJ (2013). A Kinetic-Based Model of Radiation-Induced Intercellular Signalling. PLOS ONE.

[10] Rühm W (2016). Dose-rate effects in radiation biology and radiation protection. Annals of the ICRP.

[11] Nelson JM, Todd PW, Metting NF (1984). Kinetic differences between fed and starved Chinese hamster ovary cells. Cell Tissue Kinet..

[12] Matsuya Y (2017). Modeling cell survival and change in amount of DNA during protracted irradiation. J. Radiat. Res..

[13] Pawlik TM, Keyomarsi K (2004). Role of cell cycle in mediating sensitivity to radiotherapy. Int. J. Radiat. Oncol. Biol. Phys..

[14] Sinclair WK, Morton RA (1966). X-ray sensitivity during the cell generation cycle of cultured Chinese hamster cells. Radiat. Res..

[15] Mitchell JB, Bedford JS, Bailey SM (1979). Dose-rate effects in mammalian cells in culture: III. Comparison of cell killing and cell proliferation during continuous irradiation for six different cell lines. Radiat. Res..

[16] Zips, D. Tumour growth and response to radiation. In: Joiner, M., van der Kogel, A., (eds). Basic Clinical Radiobiology. 4th edn. London: Hodder Arnold; 78–101 (2009).

[17] Hawkins RB (1996). A microdosimetric-kinetic model of cell death from exposure to ionizing radiation of any LET, with experimental and clinical applications. Int. J. Radiat. Biol..

[18] Inaniwa T, Suzuki M, Furukawa T, Kase Y, Kanematsu N (2013). Effects of dose-delivery time structure on biological effectiveness for therapeutic carbon-ion beams evaluated with microdosimetric kinetic model. Radiat. Res..

[19] Brenner DJ (2008). The linear–quadratic model is an appropriate methodology for determining isoeffective doses at large doses per fraction. Semin. Radiat. Oncol..

[20] Hawkins RB, Inaniwa T (2013). A microdosimetric-kinetic model for cell killing by protracted continuous irradiation including dependence on LET I: repair in cultured mammalian cells. Radiat. Res..

[21] Chalmers A, Johnston P, Woodcock M, Joiner M, Marples B (2004). PARP-1, PARP-2, and the cellular response to low doses of ionizing radiation. Int. J. Radiat. Oncol. Biol. Phys..

[22] ICRU. Microdosimetry. Report 36. International Commission on Radiation Units and Measurements. Bethesda: MD (1983).

[23] Tobias, C. A., Blakely, E. A. & Ngo, F. Q. H. The repair-misrepair model of cell survival. In: Meyn, R. E., Withers, H. R. (eds). Radiation Biology in Cancer Research. New York, Raven, 195–230 (1980).

[24] Curtis SB (1986). Lethal and potentially lethal lesions induced by irradiation: A unified repair model. Radiat. Res..

[25] Sato T, Furusawa Y (2012). Cell Survival Fraction Estimation Based on the Probability Densities of Domain and Cell Nucleus Specific Energies Using Improved Microdosimetric Kinetic Models. Radiat. Res..

[26] Bopp C (2016). Adaptation of the microdosimetric kinetic model to hypoxia. Phys. Med. Biol..

[27] Okamoto H (2011). Relation between lineal energy distribution and relative biological effectiveness for photon beams according to the microdosimetric kinetic model. J. Radiat. Res..

[28] Manganaro L, Cirio R, Dalmasso F, Monaco V, Sacchi R (2017). A Monte Carlo approach to the Microdosimetric Kinetic Model to account for dose rate time structure effects in ion beam therapy with application in treatment planning simulations. Phys. Med. Biol..

[29] Sato, T., Masunaga, S., Kumada, H. & Hamada, N. Microdosimetric Modeling of Biological Effectiveness for Boron Neutron Capture Therapy Considering Intra- and Intercellular Heterogenicity in ^10^B Distribution. *Sci. Rep*., **8**, Ariticle number 988 (2018). 10.1038/s41598-017-18871-0 PMC5772701 29343841

[30] Famulari G, Pater P, Enger S (2017). Microdosimetry calculations for monoenergetic electrons using Geant4-DNA combined with a weighted track sampling algorithm. Phys. Med. Biol..

[31] International Atomic Energy Agency (IAEA). Absorbed dose determination in photon and electron beams. An International Code of Practice. Technical Reports Series No. 277, Vienna (1987).

[32] Tukey JW (1949). Comparing Individual Means in the Analysis of Variance. Biometrics.

[33] Mothersill C, Seymour CB (1986). Effect of lactate on the recovery of CHO-KI cells from gamma radiation damage. Acta Radiologica: Oncology.

[34] Mao. Z, Bozzella M, Seluanov A, Gorbunova V (2008). DNA repair by nonhomologous end joining and homologous recombination during cell cycle in human cells. Cell cycle.

[35] Bird RP, Rohrig N, Colvett RD, Geard CR, Marino SA (1980). Inactivation of synchronized Chinese hamster V79 cells with charged-particle track segments. Radiat. Res..

[36] Fertil B, Dertinger H, Courdi A, Malaise EP (2012). Mean inactivation dose: A useful concept for intercomparison of human cell survival curves. Radiat. Res..

[37] ICRU, Quantitative concepts and dosimetry in radobiology, Report No. 30, International Commission on Radiation Units and Measurements, Washington, D. C. (1979).

[38] Metting NF, Braby LA, Roesch WC, Nelson JM (1985). Dose-rate evidence for two kinds of radiation damage in stationary-phase mammalian cells. Radiat. Res..

[39] Thacker J, Stretch A (1985). Responses of 4 X-ray-sensitive CHO cell mutants to different radiations and to irradiation conditions promoting cellular recovery. Mutat. Res..

[40] Jaffe DR, Haraf D, Schwartz JL, Weichselbaum RR, Diamond AM (1990). Radioresistant derivatives of an X-ray-senstive CHO cell line exhibit distinct patterns of sensitivity to DNA-damaging agents. Carcinogenesis.

[41] Weyrather WK, Ritter S, Scholz M, Kraft G (1990). RBE for carbon track-segment irradiation in cell lines of differing repair capacity. Int. J. Radiat. Biol..

[42] Tinganelli, W. Influence of LET and oxygen status on cell survival and adhesion molecule expression. PhD Thesis. Technische Universität (2012).

[43] Ma NY, Tinganelli W, Maier A, Durante M, Kraft-Weyrather W (2013). Influence of chronic hypoxia and radiation quality on cell survival. J. Radiat. Res..

[44] Kreder NC (2004). Cellular response to pulsed low-dose rate irradiation in X-ray sensitive hamster mutant cell lines. J. Radiat. Res..

[45] Falck J, Petrini JH, Williams BR, Lukas J, Bartek J (2002). The DNA damage-dependent intra–S phase checkpoint is regulated by parallel pathways. Nature genetics.

[46] Bedford JS, Mitchell JB (1973). Dose-rate effects in synchronous mammalian cells in culture. Radiat. Res..

[47] Lee H, Larner JM, Hamlin JL (1997). A p53-indipendent damage-sensing mechanism that functions as a checkpoint at the G1/S transition in Chinese hamster ovary cells. Proc. Natl. Acad. Sci. USA.

[48] Kastan MB, Lim DS (2000). The many substrates and functions of ATM. Nature reviews Molecular cell biology.

[49] Reddy NMS, Lange CS (1989). Similarities in the repair kinetics of sublethal and potentially lethal X-ray damage in log phase Chinese hamster V79 cells. Int. J. Radiat. Biol..

[50] Hall, E. J. & Giaccia, A. J. Time, Dose, and Fractionation in Radiotherapy In: Hall, E. J. & Giaccia, A. J. Radiobiology for the Radiologist 6th ed. Philadelphia: Lippincott Williams & Wilkins, 378–379 (2006).

[51] Matsuya Y, Kimura T, Date H (2017). Markov chain Monte Carlo analysis for the selection of a cell-killing model under high-dose-rate irradiation. Med. Phys..

[52] Wang H (2001). Efficient rejoining of radiation- induced DNA double-strand breaks in vertebrate cells deficient in genes of the RAD52 epistasis group. Oncogene.

[53] Hufnagl A (2015). The link between cell-cycle dependent radiosensitivity and repairpathways: A model based on the local, sister-chromatid conformationdependent switch between NHEJ and HR. DNA Rep..

[54] McMahon SJ (2012). A computational model of cellular response to modulated radiation fields. Int. J. Radiat. Oncol. Biol. Phys..

[55] McMahon, S. J., Schuemann, J., Paganetti, H. & Prise, K. M. Mechanistic Modelling of DNA Repair and Cellular Survival Following Radiation-Induced DNA Damage. *Scientific Reports*, **6** (2016). 10.1038/srep33290 PMC5022028 27624453

[56] Mitchell JB, Bedford JS, Bailey SM (1979). Dose-rate effects on the cell cycle and survival of S3 HeLa and V79 cells. Radiat. Res..

[57] Short C, Kelly J, Mayes CR, Woodcock M, Joiner MC (2001). Low-dose hypersensitivity after fractionated low-dose irradiation *in vitro*. Int. J. Radiat. Biol..

[58] Vilenchik MM, Knudson AG (2000). Inverse radiation dose-rate effects on somatic and germ-line mutations and DNA damage rates. Proc. Natl. Acad. Sci. USA.

[59] Ghita M (2015). Impact of fractionation on out-of-field survival and DNA damage responses following exposure to intensity modulated radiation fields. Phys. Med. Biol..

[60] Terashima S, Hosokawa Y, Tsuruga E, Mariya Y, Nakamura T (2017). Impact of time interval and dose rate on cell survival following low-dose fractionated exposures. J. Radiat. Res..

